# Radiobiological Implications of Nanoparticles Following Radiation Treatment

**DOI:** 10.1002/ppsc.201900411

**Published:** 2020-03-03

**Authors:** Reem Ahmad, Giuseppe Schettino, Gary Royle, Miriam Barry, Quentin A. Pankhurst, Olivier Tillement, Ben Russell, Kate Ricketts

**Affiliations:** ^1^ Division of Surgery and Interventional Science University College London Charles Bell House, 43–45 Foley Street London W1W 7JN UK; ^2^ Medical Radiation Science Group National Physical Laboratory Hampton Road Teddington Middlesex TW11 0LW UK; ^3^ Department of Medical Physics and Bioengineering University College London Malet Place Engineering Building, Gower Street London WC1E 6BT UK; ^4^ Radiation and Medical Physics Group Faculty of Engineering and Physical Sciences University of Surrey 388 Stag Hill Guilford GU2 7XH UK; ^5^ Healthcare Biomagnetics Laboratory University College London 21 Albemarle Street London W1S 4BS UK; ^6^ Institut Lumière Matière Université Claude Bernard Lyon 1 CNRS UMR 5306 Villeurbanne 69622 France; ^7^ Nuclear Metrology Group National Physical Laboratory Hampton Road Teddington Middlesex TW11 0LW UK

**Keywords:** cancer therapy, nanoparticle‐enhanced radiotherapy, radiation therapy, radiosensitization

## Abstract

Materials with a high atomic number (Z) are shown to cause an increase in the level of cell kill by ionizing radiation when introduced into tumor cells. This study uses in vitro experiments to investigate the differences in radiosensitization between two cell lines (MCF‐7 and U87) and three commercially available nanoparticles (gold, gadolinium, and iron oxide) irradiated by 6 MV X‐rays. To assess cell survival, clonogenic assays are carried out for all variables considered, with a concentration of 0.5 mg mL^−1^ for each nanoparticle material used. This study demonstrates differences in cell survival between nanoparticles and cell line. U87 shows the greatest enhancement with gadolinium nanoparticles (2.02 ± 0.36), whereas MCF‐7 cells have higher enhancement with gold nanoparticles (1.74 ± 0.08). Mass spectrometry, however, shows highest elemental uptake with iron oxide and U87 cells with 4.95 ± 0.82 pg of iron oxide per cell. A complex relationship between cellular elemental uptake is demonstrated, highlighting an inverse correlation with the enhancement, but a positive relation with DNA damage when comparing the same nanoparticle between the two cell lines.

## Graphical abstract

This study investigates the enhancement effect of nanoparticles combined with radiotherapy, relating both cell survival and DNA damage to cellular elemental uptake. Different nanoparticles and cancer cells are used, where in all cases, nanoparticles cause a level of enhancement. The complexity in enhancement with increasing uptake can be seen, highlighting different mechanisms contributing to the enhancement effect.

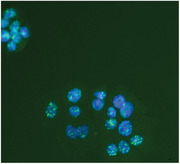

## 1 Introduction

It has been shown over the years that nanoparticles (NPs) can be used to locally enhance the level of dose deposition,^[^ ^1^ ^]^ and even in some instances cause tumors to be more sensitive to the damaging effects of ionizing radiation.^[^ ^2^ ^]^ Although this form of treatment has shown much promise, it is not currently used clinically due to the number of variables that need to be investigated to control and optimize the effect. Various groups have investigated different aspects, such as varying NP size or radiation beam energy. Even with knowledge from these studies, there is still a considerable amount of variability in reported findings. Differences are caused by the diversity in cell lines, NPs with their respective coatings, incubated NP concentrations, incubation times, irradiation parameters, as well as the assays used to demonstrate the effects. This has led to variations in the results, where significant enhancements of a factor of 25 were shown by Rahman et al., with Aurovist 1.9 nm gold nanoparticles (AuNPs) at a concentration of 1 mm with bovine aortic endothelial cells (BAEC) and 80 kV X‐rays,^[^ ^3^ ^]^ compared to smaller enhancements shown by Chithrani et al., where they synthesized 50 nm AuNPs at a concentration of approximately 1 nm in HeLa cells and found an enhancement factor of 1.17 with 6 MV X‐rays.^[^ ^4^ ^]^ As well as this, there are also differences in protocols between research groups, in both maintenance of cells and assays reported.

A review by Her et al. reported on the different mechanisms associated with NP‐enhanced radiotherapy, where the overall effect is a combination of physical, chemical, and biological mechanisms.^[^ ^5^ ^]^ In terms of the physical mechanisms, this was attributed to an increase in secondary electrons, where differences in NP size and composition can first lead to differences in interactions with the NPs, but also the number of electrons produced with sufficient energy to carry out further ionizations.^[^ ^6^ ^]^ Due to these physical mechanisms, it was initially predicted that NP‐enhanced radiotherapy would only be effective with low energy X‐rays due to difference in mass energy absorption coefficients between soft tissue and high‐Z materials, which decreased with increasing incident X‐ray energy. However, following both in vivo and in vitro experiments, significant enhancements were shown with higher energies, demonstrating that other mechanisms were involved in NP‐enhanced radiotherapy. Therefore, other mechanisms were investigated, such as chemical mechanisms. It has been suggested that this involves NPs chemically sensitizing DNA to the damaging effects of radiation, but also increasing radical formations due to the incident radiation activating the surface atoms of NPs.^[^ ^7^ ^]^ Finally, biological mechanisms were studied, reported as an increase in reactive oxygen species (ROS) formation,^[^ ^8^ ^]^ oxidative stress,^[^ ^9^ ^]^ inhibition of DNA repair,^[^ ^10^ ^]^ and changes to the cell cycle.^[^ ^11^ ^]^

To further understand the impact of introducing NPs into a cell, two different variables were considered, i) NP material and ii) cell type, all irradiated with 6 MV X‐rays. Focus was directed on these variables, as the relationship between NP material and cell type has not been characterized in terms of enhancement effect related to cellular uptake, where it has been theorized that an increase in cellular uptake would correlate with a higher enhancement effect. Findings from this work were related to the possible mechanisms that may have regulated any observed enhancement.

As the NP material can affect the radiosensitization effect, three commercially available NP types were investigated. The first were spherical AuNPs (Aurovist, Nanoprobes Inc, NY, USA; mean diameter 1.9 ± 0.6 nm, lot number 33C867), with a thiol coating.^[^ ^12^ ^]^ These NPs were used by Hainfeld et al. in the first study demonstrating in vivo NP‐enhanced radiation therapy. Second were spherical gadolinium‐based NPs (GdNPs) (AGuIX, NH TherAguix, France; mean diameter 3.0 ± 1.5 nm, batch number 2019‐01a), comprising a polysiloxane matrix with cyclic chelates of gadolinium.^[^ ^13^, ^14^, ^15^ ^]^ These NPs have recently completed a phase I clinical trial and are currently in a phase II trial (NANORAD2).^[^ ^16^ ^]^ Finally, spherical iron oxide NPs (IONPs) (RCL‐01, Resonant Circuits Limited, UK; mean diameter 140 ± 4 nm, polydispersity index 0.25 ± 0.02, batch number 2018–151) were considered.^[^ ^17^ ^]^ IONPs have been used in cancer therapy through magnetic hyperthermia, where the NPs produce heat when exposed to a high‐frequency alternating magnetic field.^[^ ^18^ ^]^ Although IONPs have not been explicitly investigated in NP‐enhanced radiotherapy, they have undergone several in vitro studies for hyperthermia applications, where they have demonstrated cellular uptake.^[^ ^19^ ^]^ It was therefore of interest to determine if these larger‐sized NPs would demonstrate similar enhancements to the other NPs considered, when combined with radiotherapy. All NP materials have a sufficiently high atomic number to observe the predicted radiosensitization effect.^[^ ^20^, ^21^ ^]^ For all NP materials, the same incubation time (24 h) and concentration of 0.5 mg mL^−1^ was used, such that comparisons could be made. This concentration was shown by Jain et al. to be effective for radiosensitization,^[^ ^22^ ^]^ when using Aurovist 1.9 nm AuNPs.

The other variable considered was the cell type, which could demonstrate in which cases NPs may be most beneficial. Two cell types were investigated, MCF‐7, a human breast adenocarcinoma cell line, and U87, a human glioblastoma cell line. Both of these cell lines are well characterized and extensively studied within the literature. They demonstrate two different cancer types, where differences in cellular uptake and radiation response were expected; therefore, it allows for trends to be identified across the two cell lines and the different NPs considered. It has been shown in the literature that depending on the cell type, differences in NP uptake can occur due to characteristics of the microenvironment, affecting NPs internalized within the cell.^[^ ^23^ ^]^ Other factors such as the surface‐to‐volume ratio can affect uptake, as a larger ratio increases the probability of interacting with cellular receptors for uptake.^[^ ^24^ ^]^ Differences in uptake across NPs and cell lines were demonstrated by Dos Santos et al., where they investigated the level of uptake in five different cell lines, with negatively charged carboxylated polystyrene (PS–COOH) NPs ranging from 40–500 nm to micrometer‐sized objects (1 and 2 µm).^[^ ^25^ ^]^ At a concentration of 20 µg mL^−1^ (24 h incubation), they demonstrated a decrease in cellular uptake with increasing NP size across all cell lines tested. Another aspect is that different cells will respond to radiation differently, where some are more radioresistant, in which case the use of NPs is of particular interest, as it may radiosensitize the cells. The reason for differences in radiosensitivity between cells is not fully understood but thought to be due to factors such as differences in ability to repair damage.^[^ ^26^ ^]^

These studies have demonstrated the difficulties in assessing the most optimum setup for NP‐enhanced radiotherapy, due to the variability in cell type, NP, and beam characteristics considered. A cooperative of stakeholders reported recommendations to standardize reporting on the efficacy of NP‐enhanced radiotherapy, highlighting metrics such as NP cellular uptake and cell survival (using fitting parameters α and β obtained from the linear‐quadratic (LQ) model), as well as quoting an enhancement ratio at a dose level of 2 Gy.^[^ ^27^, ^28^ ^]^ These reports also highlighted that although the α/β ratio would indicate changes in radiosensitivity due to the addition of NPs, no study has currently incorporated this metric within their analysis of the enhancement effect. An interesting aspect of this is to compare findings with the literature on samples irradiated with higher linear energy transfer (LET) radiation, such as protons, which show an increased relative biological effectiveness (RBE) due to more densely ionizing incident radiation.^[^ ^29^ ^]^ By comparing with in vitro studies that irradiate samples with protons, it is possible to determine if the addition of NPs can alter the α/β ratio of X‐ray irradiations to be comparable to radiation with a higher LET (protons). Finally, the quantification of DNA damage in the form of immunofluorescence staining has been identified as a key factor regulating radiation response, which needs to be quantified in terms of NP‐enhanced radiotherapy. Therefore, this study aims to unify these key metrics in a parameterized study, considering the effect of NP type on cell line, relating cell survival and DNA damage to cellular uptake. This was demonstrated through both clonogenic assays, quantifying cell survival post‐irradiation, and the 53BP1 foci formation assay, which quantifies DNA double‐strand break (DSB) repair through the marker 53BP1.^[^ ^30^, ^31^ ^]^ Both results were compared to uptake measurements using inductively coupled plasma mass spectrometry (ICP‐MS). The results from this study offer an understanding of the biological impact of using NPs though an in vitro study, comparing variability in biological effect across both cell line and NP types and relating findings with cellular uptake.

## 2 Results

### 2.1 Nanoparticle Cytotoxicity

**Figure 1:**
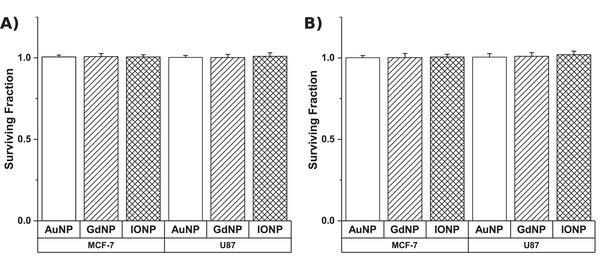
Cytotoxic effect of nanoparticles on clonogenic survival, carried out in triplicate, following 24‐h exposure to a concentration of A) 0.2 mg mL^−1^ and B) 0.5 mg mL^−1^ of each respective nanoparticle material. Surviving fractions were normalized to untreated control cells in each experiment.

The effect of a 24 h exposure to NPs was assessed through the clonogenic assay as shown in **Figure** **1**. It can be seen that for both concentrations considered, all three NPs has no impact on the cells ability to produce colonies for either cell line considered.

### 2.2 Nanoparticle Uptake

**Figure 2:**
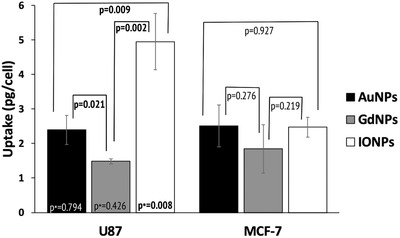
Uptake measurements, carried out in triplicate, determined using mass‐spectrometry where results were quoted as pg of gold, gadolinium, and iron oxide per cell. *p*‐Values represent comparisons between the three NP types for each cell line, whereas p* values represent comparisons of the same NP across both cell lines.

Using ICP‐MS, differences in cellular uptake were reported between cell lines and nanoparticle types following a 24 h incubation period. **Figure** **2** shows that the highest uptake was with U87 cells and IONPs. Significance was demonstrated between all NPs for U87 cells, whereas with MCF‐7 cells, no significant difference in cellular uptake was shown between the NPs considered. When comparing the uptake for the same NP, the only significance demonstrated was with IONPs, whereas no significant differences were shown with the other NPs between the two cell lines.

### 2.3 Clonogenic Variations Due to the Addition of NPs

**Figure 3:**
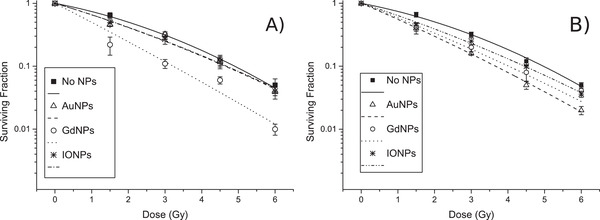
Cell survival curves for A) U87 and B) MCF‐7, where for both cell lines, cells + AuNPs, cells + GdNPs or cells + IONPs were assessed, as well as a control of cells alone. Samples were irradiated with X‐rays from a 6 MV linac in triplicate. NPs added at a concentration of 0.5 mg mL^−1^ and incubated for 24 h.

**Figure****3** demonstrates the cell survival with and without NPs for both cell lines, where the α/β ratios were determined for all curves and significant changes were shown due to the addition of NPs, for all types considered, with both cell lines (**Table** **1**). For U87 cells, the α/β ratios were 86.1 ± 41.5 Gy (*p* = 0.029), 20.5 ± 8.73 Gy (*p* = 0.047), and 20.5 ± 8.90 Gy (*p* = 0.049) for GdNPs, AuNPs, and IONPs, respectively, compared to a ratio of 6.02 ± 1.19 Gy for cells alone. For MCF‐7 cells, ratios were 39.6 ± 15.6 Gy (*p* = 0.021), 39.3 ± 17.4 Gy (*p* = 0.030), and 22.9 ± 9.91 Gy (*p* = 0.044) for GdNPs, AuNPs, and IONPs, respectively, compared to a ratio of 6.15 ± 1.32 Gy for cells alone, where *p*‐values quoted compare samples with NPs to the control without NPs. An increase in the α/β ratio signifies that tumor response is less dependent on the dose per fraction, therefore a lower dose can be used.^[^ ^32^ ^]^ An interesting point was the significant decrease in β for U87 cells with GdNPs, where a decrease in β indicates a smaller proportion of repairable cell damage. Having shown a significance between NPs and the control (Table 1, *p*‐value_1_), comparisons were made for the same NP between the two cell lines (Table 1, *p*‐value_5_), where no significance was shown for all NPs considered, demonstrating that the NPs had a biological effect independent of the cell line considered.

**Table 1:** Fitting parameters, alpha and beta for each sample, along with the ratios and *p*‐values, highlighting significance between control and NP samples, based on a *t*‐test

Sample	α [Gy^−1^]	β [Gy^−2^]	Ratio [Gy]	*p*‐Value_1_	*p*‐Value_2_	*p*‐Value_3_	*p*‐Value_4_	p‐value_5_
U87 cells	0.259 ± 0.030	0.043 ± 0.007	6.02 ± 1.19					
U87 cells + AuNPs	0.404 ± 0.086	0.020 ± 0.007	20.5 ± 8.73	**0.047**				0.170
					**0.049**			
U87 cells + GdNPs	0.689 ± 0.173	0.008 ± 0.003	86.1 ± 41.5	**0.029**				0.143
						0.999		
U87 cells + IONPs	0.401 ± 0.044	0.020 ± 0.008	20.5 ± 8.90	**0.049**				0.772
							**0.049**	
MCF‐7 cells	0.256 ± 0.035	0.042 ± 0.007	6.15 ± 1.32					
MCF‐7 cells + AuNPs	0.575 ± 0.029	0.015 ± 0.006	39.3 ± 17.4	**0.030**				
					0.983			
MCF‐7 cells + GdNPs	0.520 ± 0.049	0.013 ± 0.005	39.6 ± 15.6	**0.021**				
						0.229		
MCF‐7 cells + IONPs	0.432 ± 0.042	0.019 ± 0.009	22.9 ± 9.91	**0.044**				
							0.193	

The first *p*‐value represents comparisons between each respective NP and the control without NPs, whereas the second compares AuNPs and GdNPs, the third AuNPs and IONPs, the fourth GdNPs and IONPs, and the fifth compares the same NP between the two cell lines considered.

### 2.4 Enhancement Factors

**Figure 4:**
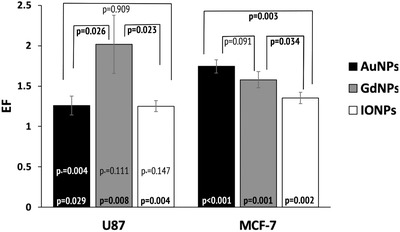
Enhancement factors calculated from clonogenic cell survival fittings comparing doses needed for equivalent cell survival at a dose of 2 Gy for the control sample. The *p*‐value within the bar represents comparisons between each respective NP and the control, the p_*_ value represents the comparison of the same NP between the two cell lines, whereas the values above the bars compare the different NP types for each cell line.

For U87 cells, all NPs considered caused an increase in the level of cell kill, where the highest was with GdNPs showing an EF of 2.02 ± 0.36 (*p* = 0.008), as shown in **Figure** **4**, where it was 1.27 ± 0.12 (*p* = 0.020) and 1.26 ± 0.07 (*p* = 0.004) for AuNPs and IONPs, respectively. With MCF‐7 cells, the highest EF was 1.74 ± 0.08 (*p* < 0.001) with AuNPs, compared to 1.58 ± 0.10 (*p* = 0.001) and 1.35 ± 0.07 (*p* = 0.002) for GdNPs and IONPs, respectively. In terms of comparing enhancements between NPs, with U87 cells, there were significant differences between AuNPs and GdNPs (*p* = 0.026), then GdNPs and IONPs (*p* = 0.023). With MCF‐7 cells, significant differences were shown between AuNPs and IONPs (*p* = 0.003), then GdNPs and IONPs (*p* = 0.034). When comparing the same NP between the two cell lines, a significance in EF was only demonstrated with AuNPs (p_*_ = 0.004).

### 2.5 DNA DSB Damage

**Figure 5:**
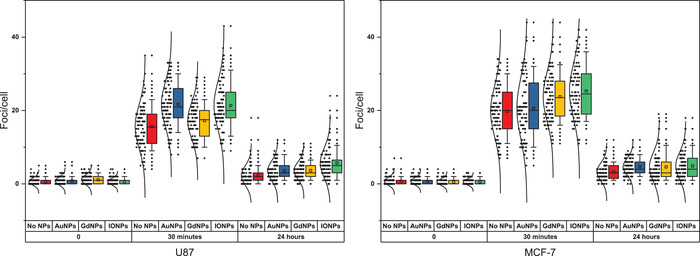
Quantitative analysis considering the effect of AuNPs, GdNPs, and IONPs on DSBs formation in a) U87 cells (left) and b) MCF‐7 cells (right). Samples were exposed to 1 Gy using 6 MV X‐rays, quantifying foci per cell. The lower part of the boxes indicates the first quartile, dividing line shows the median, square shows the mean, and top line shows the third quartile. The lower and upper ends of the whisker indicate 10th and 90th percentile. For each data point, 50 cells were counted for three independent replicates, where the individual counts are depicted to the left of each respective box, with a normal distribution.

**Figure 6:**
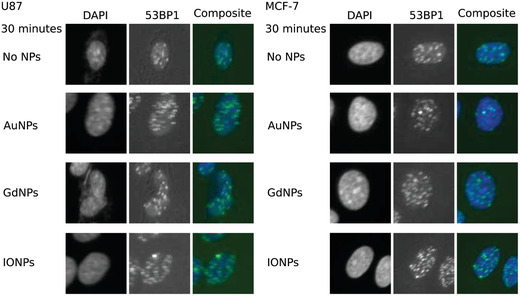
Immunofluorescence performed for 53BP1 foci analysis using a CellInsight CX5High Content Screening Platform (×20 magnification) to image the samples for both U87 (left) and MCF‐7cells (right).

**Figure****5** demonstrates DNA DSB damage following irradiation, where changes in foci were quantified at two time points, with and without NPs. Examples of images of cells following immunofluorescence staining are shown in **Figure** **6**. From Figure 5, it can be seen that the addition of NPs causes changes in the number of foci per cell at both time points, where considering the means, the differences were shown to be statistically significant in all cases tested. For both cell lines, the greatest residual damage was shown with IONPs when considering the third quartile (108% and 40% greater and the control for U87 and MCF‐7, respectively, compared to 67% and 20% for both AuNPs and GdNPs). Interestingly, however, when considering immediate damage with U87 (30‐min time point), the greatest damage was shown with AuNPs, although a significant portion remained unrepaired (24‐h time point); the NPs that showed the least repair were IONPs. Considering the 24 h data, it can be seen from Figure 5 that for U87 cells, AuNPs and GdNPs show comparable results in terms of the first and third quartiles and the median, with similar distributions shown, whereas IONPs showed the greatest spread and highest median value. With MCF‐7 cells, the means were the same; however, the spreads differed, leading to the median value of GdNPs being lower than both AuNPs and IONPs. As with U87 cells, IONPs showed the greatest spread for MCF‐7 cells. These deviations from a Gaussian distribution can indicate the presence of a subpopulation of cells that have responded differently to the treatment of ionizing radiation combined with NPs.

### 2.6 Effect of Cellular Elemental Uptake on Enhancement

**Figure 7:**
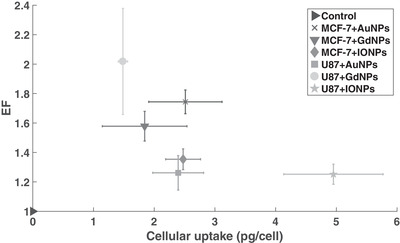
Comparisons between the enhancement factors and cellular elemental uptake for each respective setup, where control represents any cell without NPs, where the uptake would be 0 and there would be no enhancement; therefore, the EF would be 1. The *p*‐values comparing the cellular elemental uptake for the same NP across the two cell lines were 0.794, 0.426, and 0.008 for Au, Gd, and IONPs, respectively. Comparing the EF for the same NP across the two cell lines, the *p*‐values were 0.005, 0.111, and 0.147 for Au, Gd, and IONPs, respectively.

**Figure****7** shows that for the cell lines and NPs used, at an incubation concentration of 0.5 mg mL^−1^, there was a non‐linear inverse relationship between cellular elemental uptake and enhancement. Although significance was shown between cells with no NPs and cells with NPs for both cell lines (Figure 4), the level of enhancement did not increase with elemental uptake. An example of this can be seen with U87 cells, where IONPs showed the highest elemental uptake but the lowest level of enhancement. It should be noted, however, that elemental uptake does not directly correlate to NP uptake due to differences in structures, molecular weights, compositions, and sizes of NPs considered, where the highest elemental uptake may not correspond to the highest number of NPs internalized. Therefore, only the total amount of material per cell was considered, rather than converting into approximate number of NPs per cell. Figure 7 consequently demonstrates the relationship between the total amount of material and the enhancement effect, through the EF, suggesting the presence of an optimum uptake. The control represents any cell that was not exposed to NPs, where, by definition, there would be no enhancement or uptake to measure.

**Figure 8:**
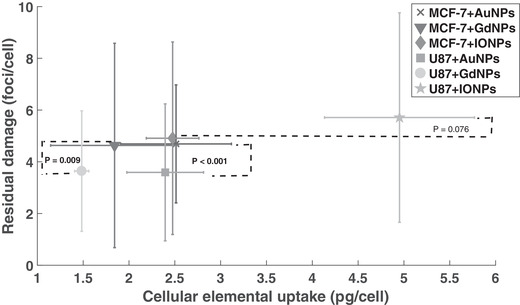
Comparisons between the residual DNA damage and cellular elemental uptake for each respective setup. The *p*‐values represent comparisons between the two cell lines for the same NP.

When comparing the same NP between the two cell lines, it was interesting to note that significance was demonstrated in EF between AuNPs, but not in elemental uptake, whereas with IONPs, significance was shown in elemental uptake but not in the EF. The same trends were shown in the residual damage (**Figure** **8**) for both IONPs and AuNPs; with GdNPs, however, no significance was shown in elemental uptake or EF, but was shown in terms of residual damage.

## 3 Discussion

Considering the clonogenic assay work, it was evident from the results that the addition of NPs created a significant decrease in cell survival (Figure 4). Although this decrease was observed for both cell lines and both NP types, the rate of decrease differed between the two variables. From Table 1, it was evident that the addition of NPs both increased the alpha value, indicating more cells killed per Gy and decreased the beta value, suggesting a smaller proportion of repairable cell damage. Together, these led to higher statistically significant α/β ratios for both cell lines and NPs. For both cell lines, the greatest ratio increase was with GdNPs. It was found that the control value for U87 cells (no NPs) reported in this study, was comparable to those quoted in the literature for cells irradiated with 6 MV X‐rays,^[^ ^33^, ^34^, ^35^ ^]^ where values ranged between 5 and 10 Gy and our value was 6.02 ± 1.19 Gy. The MCF‐7 control (no NPs) was also comparable to those in the literature which ranged from 2 to 10 Gy.^[^ ^36^, ^37^ ^]^

When comparing the ratios of the same NPs between the two cell lines, no significance was demonstrated for all NPs considered, highlighting that the NPs had an effect on cell kill irrespective of the cell line considered. In terms of enhancement, a statistically significant increase in enhancement was demonstrated with all NPs and cell lines (Figure 4). This correlated with statistically significant changes in the formation of DSBs (Figures 5 and 6). An example of this was with U87 cells and GdNPs, where the β component was significantly decreased in the cell survival and the residual damage showed a significant increase. In terms of the residual damage, however, the greatest change in foci per cell was shown with IONPs for U87 cells, which showed the lowest ratio increase in terms of cell survival. This highlights that the enhancement due to NPs is complex and not only due to an increase in DNA damage.

Similar findings in DNA damage were reported by Taggart et al., where they also considered the use of a breast and glioma cell line (MDA‐MB‐231 and T98G, respectively). With this study, they only considered one NP type, the same commercial AuNP used in this study; however, they irradiated their samples using 225 kVp X‐rays, rather than the 6 MV linac used in this study. They demonstrated statistically significant increase in DNA damage with the breast cancer cell line; however, with the glioma cell line, they showed a statistically significant decrease in DNA damage.^[^ ^38^ ^]^

Another factor for the treatment was the radiation type used, as it is known that when irradiating with higher LET radiation, the survival curve is steeper, which indicates less repaired damage. The linear survival curve with particle radiation is due to an increasing alpha value with increasing LET,^[^ ^39^ ^]^ indicating a higher α/β ratio than X‐rays, where a point of interest was to identify if the addition of NPs to cells irradiated by X‐rays could show comparable damage to that observed with higher LET radiation. From the work of Chaudhary et al., the α/β ratio for U87 cells irradiated by protons at an LET of 11.9 keV µm^−1^ was approximately 10 Gy.^[^ ^40^ ^]^ Our findings show higher values than this for both types of NPs (2.0, 8.6, and 2.0 times higher for Au, GdNPs, and IONPs, respectively), indicating a more linear survival curve, similar to that seen with protons, but in our case, even steeper than protons for both NPs. This suggests that the addition of NPs increases the biological effectiveness of the treatment, offering comparable damage to that of higher LET radiation.

In comparison to the study by Stefancikova et al., which also used GdNPs, a previous formulation of the commercialized AGuIX NPs, and U87 cells irradiated with X‐rays (cobalt source compared to measurements with a linac in this study), at a concentration of 0.5 mg mL^−1^ (12 h incubation compared to 24 h used in our study), there was a difference in the observed EF, where they report a factor of 1.23, whereas this study found a value of 2.02.^[^ ^41^ ^]^ A possible reason for this difference is that although both considered GdNPs and the same cell line, incubation times differed. The longer incubation time would have led to different number of NPs internalized within the cell, resulting in a different EF; however, this study did not quote the average amount of Gd taken up within a cell.

For comparisons to be made between the NP types and cell lines used, quantification of the cellular elemental uptake was needed (Figure 2). It was theorized that a higher level of elemental uptake would correlate with a higher enhancement effect, where only elemental uptake was considered rather than converting values to approximate number of NPs. This was chosen as in practice differences in molecular weights, compositions, coatings, and final size due to the protein corona would alter the number of NPs internalized within the cell. Therefore, by considering the bulk elemental material, we can make comparisons on the effect this has on the enhancement effect of cells exposed to NPs of an equal incubation concentration. Within this study, the complex nature of NP‐enhanced radiotherapy was demonstrated, where with regards to the cellular uptake of bulk NP material, it was suggested that there was an optimum level of uptake to observe the highest enhancement effect. Figure 7 shows that although the highest level of elemental uptake was demonstrated with iron oxide, it did not correlate with the highest enhancement factor. Similarly, when considering the same NP, GdNPs with U87 cells showed the highest biological effect in terms of EF yet had a lower level of elemental uptake compared with MFC‐7 cells; however, no significance was demonstrated in terms of elemental uptake between these cell lines for GdNPs. In contrast, significance was shown in elemental uptake for IONPs, where a higher enhancement was shown at a lower uptake; whereas in terms of enhancement, no significance was shown between the cell lines.

Interestingly, Figure 8 shows the inverse, whereby for all NPs and cells considered, a higher level of residual damage was shown with a higher elemental uptake. From these findings, the complexity of mechanisms regulating radiobiological enhancement can be seen, where the initial enhancement could be due to the combined effect of physical, chemical, and biological mechanisms. At higher levels of elemental uptake, however, if higher elemental uptake correlated with a higher number of NPs, the effect of these mechanisms would be altered, where physical enhancement may be reduced due to secondary electrons being absorbed in neighboring NPs. Similarly, a higher number of NPs could act as ROS scavengers, reducing the amount of ROS present to damage the cell,^[^ ^42^ ^]^ thereby reducing both chemical and biological enhancement. Other cellular responses, resulting in biological mechanisms such as oxidative stress and cell cycle effects could have caused differences in the biological effect, which may not be dependent on a higher level of elemental uptake.

## 4 Conclusion

This study was able to demonstrate biological changes encountered by cells due to the presence of NPs combined with ionizing radiation. It was possible to quantify differences between both cell lines and NP types. In terms of the clonogenic assays with U87 cells, an enhancement of dose was observed, whereby a lower dose needed to be delivered (0.98 Gy with GdNPs compared to 2 Gy without NPs) to offer the same level of cell survival as that observed with cells alone at 2 Gy. This study has highlighted the complex relationship between elemental uptake and enhancement effect both in terms of cell survival and DNA damage. It was shown that when considering the same NP between the two cell lines, a higher enhancement effect was related to lower cellular elemental uptake, whereas the DNA damage increased with increasing elemental uptake. This alluded to differences in contribution from mechanisms highlighted in the literature, changing with increasing elemental uptake. Although significance in enhancement was demonstrated by comparing NP‐inoculated to NP‐free samples, significance was only demonstrated for AuNPs when comparing the same NP between the two cell lines. This therefore indicates that the NP type, elemental uptake, or cell type alone cannot explain the radiobiological enhancement effect observed with the NPs and cell lines used in this study. Further work would be to investigate different combinations of these parameters to explain the effects observed. One aspect would be to consider different incubation concentration to demonstrate if an optimum uptake can be identified. Others would be to use specific assays to highlight the different mechanisms in place to demonstrate their overall contribution to cell kill following NP‐enhanced radiotherapy.

## 5 Experimental Section

### Cell Culture

Two cell lines were investigated, MCF‐7 human breast adenocarcinoma cell line and U87 human glioblastoma cell line. Both cell lines were cultured in Minimum Essential Medium (MEM) (Fisher Scientific, UK), supplemented with 10% fetal bovine serum and 1% penicillin–streptomycin (Fisher Scientific, UK). All cells were maintained in monolayers in a tissue culture incubator at 37 °C with 5% CO_2_/95% air.

### Nanoparticles

Three nanoparticles were investigated; the first were freeze‐dried spherical AuNPs (Aurovist) with a mean diameter of 1.9 ± 0.6 nm (Nanoprobes Inc, NY, USA) that were re‐dispersed in phosphate buffered saline (PBS) (Fisher Scientific, UK) and stored at 20 °C as per the manufacturer's instructions. The second were freeze‐dried gadolinium‐based nanoparticles (AGuIX) with a mean diameter of 3 ± 1.5 nm (NH TherAguix, France). These were re‐dispersed in ultrapure water and stored at 4 °C. The third were magnetic dextran iron oxide nanoparticles (RCL‐01) in water with a mean (Z‐average) diameter of 140 ± 4 nm and a polydispersity index of 0.25 ± 0.02 (Resonant Circuits Limited, UK), stored at 4 °C. All stock solutions were diluted in culture medium before adding a concentration of 0.5 mg mL^−1^ of each respective NP material to the seeded cells.

### Nanoparticle Toxicity

To ensure the concentrations used were non‐toxic, a fixed number of cells were seeded onto six‐well plates (Sigma‐Aldrich, UK), one plate for each cell line, where two concentrations of each nanoparticle were introduced. From the literature, it was reported that a concentration of 0.5 mg mL^−1^ (24 h incubation) was not toxic; therefore, this and a lower concentration of 0.2 mg mL^−1^ were tested.^[^ ^18^ ^]^ Following the incubation period, medium and nanoparticle mixture was removed, wells were gently washed twice with PBS, and each well was filled with 5 mL of fresh medium. The plates were incubated for 10–14 days, fixed, stained, and colonies were counted and compared to control plates without NPs.

### Sample Preparation

Having confirmed the concentration of 0.5 mg mL^−1^ was not toxic, a clonogenic assay investigation was conducted in triplicate. For the irradiations, 10^5^ cells mL^−1^ were seeded onto 35 mm culture dishes (Corning, UK) 48 h prior to irradiation, to reach between 80–100% confluence on the day of irradiation. The NP samples were incubated for 24 h prior to irradiation with 1 mL medium containing NPs at a concentration of 0.5 mg mL^−1^. Medium was also changed for the control samples 24 h prior to irradiation. Following the incubation period and just prior to irradiation, NP solution was removed, the samples were gently washed twice with PBS to remove any extracellular NPs, and fresh medium was added to each dish.

### Uptake Measurements

As with previous samples, cells were seeded, and NPs were added. Following the incubation period, the NP solution was removed, and dishes were gently washed twice with PBS. Cells were trypsinized and counted with a hemocytometer to determine the total number of cells per sample. The cell suspension was centrifuged for 15 min at 1000 RPM. Pellets were then dissolved by aqua regia (three parts hydrochloric acid to one part nitric acid) and the solution was diluted with ultrapure water. Using ICP‐MS (Agilent 8800, Cheadle, UK), reference measurements were initially carried out on a known concentration of each NP type, diluted to obtain a reference curve relating the counts per second to the NP concentration.^[^ ^43^ ^]^ Each sample was then processed, and counts were related to the reference curve to determine the elemental concentration of each material per sample. Results were then reported as the mass of NP element per cell (pg per cell).

### Irradiation

**Figure 9:**
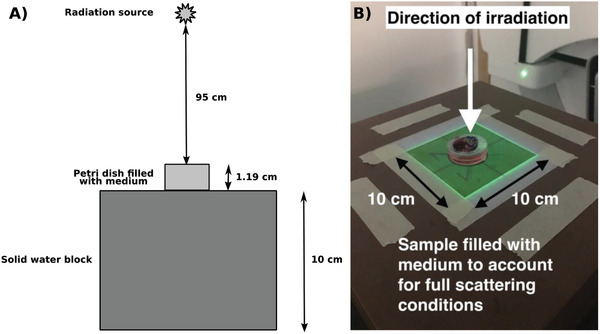
Schematic demonstrating the irradiation setup with the distances between the source and the sample surface. Dose was established following TRS398 Code of Practice and scaled to the correct depth for the cells at the bottom of the petri dish using the percentage depth dose curves (A). Experimental setup for clonogenic assay irradiations using 6 MV X‐rays, where the sample was placed on blocks of solid water in a 10 × 10 cm^2^ field (B).

For clonogenic X‐ray irradiations, five dose points were investigated in triplicate, 0, 1.5, 3, 4.5, and 6 Gy, chosen to decrease the survival fraction by approximately two orders of magnitude to give a sufficient cell survival curve fitting for each cell line and nanoparticle combination, whereas the DNA damage samples were irradiated with 1 Gy. Before irradiating with X‐rays, the dishes were filled with medium, providing adequate scattering conditions. The dishes were then sealed using Parafilm M (Sigma‐Aldrich, UK) immediately before irradiation. Irradiations were carried out at the National Physical Laboratory, Teddington, UK, with a 6 MV linac (Elekta Versa HDTM), with a dose rate of 6.5 Gy min^−1^. Dishes were placed in the center of a 10 × 10 cm^2^ field (**Figure** **9**), where dose calculations were based on reference conditions using depth dose data from ionization chamber measurements for this field size. The dose output was also confirmed with ionization chamber measurements traceable to the UK primary standard. The beam uniformity was assessed using Gafchromic EBT3 film (Ashland ISP Advanced Materials, NJ, USA), where the dose variation across the sample was less than 2%.

### Clonogenic Assay

Following irradiation, each flask was washed twice with PBS, trypsinized, counted, and re‐plated onto six‐well plates and incubated for 10–14 days.^[^ ^44^ ^]^ Following this, colonies were stained with 0.4% crystal violet and counted using a Nikkon Eclipse Ti‐S inverted microscope. The plating efficiency was calculated as the ratio of the colonies formed to cells seeded. The surviving fraction (SF) was calculated as the plating efficiency of the irradiated sample divided by the plating efficiency of the unirradiated sample. The SF was plotted against the dose, where the curves were then fitted to the LQ model described by Equation (1)

(1) $$\text{SF}\;=\;{\text{e}}^{-\left(\alpha D\;+\;\beta D^{2}\right)}$$

where α and β are fitting parameters and *D* is the delivered dose. α and β values were determined from the curve fitting, carried out using OriginPro software, version 9 (OriginLab Inc., Northampton, MA), using a non‐linear least‐squares fitting procedure, weighted to the errors associated with each measurement. From this the α/β ratio was determined for each curve. The enhancement factor (EF) was determined as the ratio of dose required for NP treated cells to give same survival as cells not treated with NPs, irradiated at 2 Gy, where the ratios were determined using the SF fits (Equation (2)).

(2) $$\text{EF}\;=\frac{\;\text{Reference}\;\text{dose}\;\text{of}\;2\;\text{Gy}}{\text{Dose}\;\text{needed}\;\text{with}\;\text{Cells}+\text{NPs}\;\text{to}\;\text{achieve}\;\text{the}\;\text{same}\;\text{cell}\;\text{survival}\;\text{as}\;\text{Cells}\;\text{alone}\;\text{at}\;\text{a}\;\text{dose}\;\text{of}\;2\;\text{Gy}}$$

Statistical analysis was performed using a *t*‐test with the statistical software SPSS (IBM Corp). Significance was tested both between samples containing NPs compared to control samples and between the different NP samples.

### Foci Formation Assay

For the staining of the DNA damage marker 53BP1, samples were fixed at two timepoints, one 30 min post‐irradiation, another after 24 h, demonstrating the immediate and unrepaired damage post‐irradiation respectively, the latter being referred to as the ‘residual DNA damage' or ‘residual damage' of the samples. Medium was removed and each dish was washed twice with PBS. Cold fixing solution (methanol:acetic acid) was added to each dish and left for 30 min at 4 °C. The solution was then removed and replaced with PBS where samples were stored at 4 °C until immunofluorescence staining. In terms of the staining, samples were washed with cold PBS, permeabilized (0.5% Triton X‐100 in PBS), and blocked in 10% goat serum, 1% BSA, and 0.1% Triton X‐100 in PBS for 1 h at 37 °C in an incubator. Cells were probed with 53BP1 primary antibody raised in rabbit (Novus Biologicals, USA) at a dilution of 1:1000 and incubated at 37 °C for an hour. Samples were washed three times, then probed with goat anti‐rabbit Alexa Flour 488‐conjugatedsecondary antibody (Invitrogen, Life Technologies, USA) at a dilution of 1:1000 (samples were covered from this point due to light sensitivity), and incubated for an hour. Samples were then washed three times and counterstained with DAPI (Sigma‐Aldrich, UK) where they were then imaged using the CellInsight CX5 High Content Screening Platform (Thermo Fisher Scientific, Inc). For each data point, foci were manually randomly counted in 50 cells, where each point had triplicate experiments. Statistical significance was determined using a *t*‐test.

## Conflict of Interest

One of the authors (Q.A.P.) is a shareholder in, and part‐time advisor to, companies (Resonant Circuits Limited and Endomagnetics Limited) with commercial interests in magnetic nanoparticles. Another author (O.T.) has to disclose the patents WO2011/135101 and WO2009/053644. These patents protect the AGuIX NPs described in this publication. O.T. is an employee and shareholder of the company NH TherAguix, who are developing the AGuIX NPs.
